# Hematological and biochemical parameters of giant pandas (*Ailuropoda melanoleuca*) in captive and semi-natural environments

**DOI:** 10.1093/conphys/coad083

**Published:** 2024-02-15

**Authors:** Wenlei Bi, Songrui Liu, Michael P O’Connor, Jacob R Owens, Marc T Valitutto, Rong Hou, Dunwu Qi, Lee-Ann Collins Hayek, Fanqi Wu, Rui Ma, Jiabin Liu, Yanshan Zhou, Long Zhang, Ramana Callan, Li Luo, Wenjun Huang, Zhihe Zhang, James R Spotila

**Affiliations:** Department of Biodiversity, Earth and Environmental Science, Drexel University, 3145 Chestnut St, Philadelphia, PA 19104, USA; Sichuan Key Laboratory of Conservation Biology for Endangered Wildlife, Chengdu Research Base of Giant Panda Breeding, 1375 Panda Rd, Chengdu, Sichuan 610081, China; Sichuan Key Laboratory of Conservation Biology for Endangered Wildlife, Chengdu Research Base of Giant Panda Breeding, 1375 Panda Rd, Chengdu, Sichuan 610081, China; Department of Biodiversity, Earth and Environmental Science, Drexel University, 3145 Chestnut St, Philadelphia, PA 19104, USA; Department of Conservation, Los Angeles Zoo, Botanical Gardens, 5333 Zoo Dr Los Angeles, California, CA 90027, USA; EcoHealth Alliance, 520 Eighth Avenue, Ste. 1200, New York, NY 10018, USA; Sichuan Key Laboratory of Conservation Biology for Endangered Wildlife, Chengdu Research Base of Giant Panda Breeding, 1375 Panda Rd, Chengdu, Sichuan 610081, China; Sichuan Key Laboratory of Conservation Biology for Endangered Wildlife, Chengdu Research Base of Giant Panda Breeding, 1375 Panda Rd, Chengdu, Sichuan 610081, China; Smithsonian Institution, MRC, PO Box 37012, SI Building, Room 153, MRC 010, Washington, DC 20013, USA; Global Cause Foundation, 1002 Doe Run, Blacksburg, VA 24060, USA; Purdue University at Fort Wayne, 2101 E. Coliseum Blvd., Fort Wayne, IN, USA; Sichuan Key Laboratory of Conservation Biology for Endangered Wildlife, Chengdu Research Base of Giant Panda Breeding, 1375 Panda Rd, Chengdu, Sichuan 610081, China; Sichuan Key Laboratory of Conservation Biology for Endangered Wildlife, Chengdu Research Base of Giant Panda Breeding, 1375 Panda Rd, Chengdu, Sichuan 610081, China; Sichuan Key Laboratory of Conservation Biology for Endangered Wildlife, Chengdu Research Base of Giant Panda Breeding, 1375 Panda Rd, Chengdu, Sichuan 610081, China; Sichuan Key Laboratory of Conservation Biology for Endangered Wildlife, Chengdu Research Base of Giant Panda Breeding, 1375 Panda Rd, Chengdu, Sichuan 610081, China; Sichuan Key Laboratory of Conservation Biology for Endangered Wildlife, Chengdu Research Base of Giant Panda Breeding, 1375 Panda Rd, Chengdu, Sichuan 610081, China; Miami University, 501 E. High St, Oxford OH, USA; Sichuan Key Laboratory of Conservation Biology for Endangered Wildlife, Chengdu Research Base of Giant Panda Breeding, 1375 Panda Rd, Chengdu, Sichuan 610081, China; Sichuan Key Laboratory of Conservation Biology for Endangered Wildlife, Chengdu Research Base of Giant Panda Breeding, 1375 Panda Rd, Chengdu, Sichuan 610081, China; Sichuan Academy of Giant Panda, 1375 Panda Rd, Chengdu, Sichuan Province, China; Department of Biodiversity, Earth and Environmental Science, Drexel University, 3145 Chestnut St, Philadelphia, PA 19104, USA; Global Cause Foundation, 1002 Doe Run, Blacksburg, VA 24060, USA

**Keywords:** acclimation, biochemistry, blood parameter, complete blood count, elevation, giant panda, reference intervals, reintroduction

## Abstract

Physiological indexes like blood parameters have been widely used to monitor the health of free-roaming animals. Attempts to reintroduce one of China’s most endangered species, the giant panda (*Ailuropoda melanoleuca*), have been hampered by a lack of data on its ecology and physiology. We examined three giant pandas’ hematological and blood chemistry parameters in a soft release program and 30 captive giant pandas as controls and determined the reference intervals (RIs) for those blood parameters in the captive animals. Elevation, captivity status and the interaction of those factors were statistically significant for hematologic measures. Release pandas had significantly higher hemoglobin and hematocrit values after they moved to high elevation locations. We also found significant difference in the enzyme parameters between high and low elevation pandas such as higher aspartate aminotransferase, alanine aminotransferase, creatinine kinase, amylase and lower lactate dehydrogenase and alkaline phosphatase. Release pandas also had higher nutrition parameter values such as higher albumin, globulin and creatinine. The RI for blood parameters in our study provides a baseline to monitor the health of captive animals and forms the basis for assessing the health of free-roaming giant pandas in future reintroduction efforts.

## Teaser

We defined the reference intervals for blood parameters of giant pandas. This is the first study to reveal changes in blood parameters in giant pandas in response to changes in elevation. These data help us to plan reintroduction of captive born giant pandas.

## Introduction

The giant panda (*Ailuropoda melanoleuca*) is a unique bear species (Family Ursidae) endemic to mainland China. Unlike most other members of the Order Carnivora, giant pandas are primarily herbivorous and eat 10–38 kg of bamboo leaves, stems, and shoots per day (Schaller *et al.,*1985). In light of their unique features and endemism, pandas are considered a national treasure in China and have become an important symbol of Chinese culture. This importance led to the development of the National Conservation Project for the giant panda and its habitat, which has led to a variety of conservation initiatives, including the establishment of 67 reserves for giant pandas totaling roughly 33 600 km^2^ (Kang and Li, 2018). After nearly 50 years of conservation there are now about 1900 wild giant pandas, a 17% increase compared to the survey ten years prior (2000–2004) (Sichuan Forestry Department, 2015).

Translocation of wildlife, especially flagship species like giant pandas, can be effective in increasing not only the population of the target species but in protecting other sympatric species in the same area (Li and Pimm, 2016). Since 2005, the Chinese government has released 13 giant pandas into the wild, including three rescued and ten captive born and raised individuals (Sichuan Forestry Department, 2018). However, only one of the rescued giant pandas survived. Two of the released individuals survived more than three years, one of the captive raised bears was confirmed dead after one year, and the status of the others was unknown (Sichuan Forestry Department, 2018; Yang *et al*., 2018a). Knowledge of the physiological condition of the released giant pandas in the wild would have provided data critical to their survival. For example, blood sampling of our release pandas in large enclosures in a Nature Reserve indicated that they were sick and further analysis discovered a new Babesia parasite. Immediate treatment saved their lives, and we have added blood Babesia monitoring into our monthly health monitoring protocol (Yue *et al.,*2020).

The Chengdu Research Base of Giant Panda Breeding (Panda base) in Chengdu, China adopted an assisted soft release (ASR) method, pioneered with American black bears (*Ursus americanus*) (Kilham and Gray, 2002; Kilham and Spotila, 2021), to release captive-born giant pandas back into their native range to augment existing, small populations or restore those that have been extirpated. This method has been used with Asiatic black bears (*Ursus thibetanus*) in India (Beecham *et al.,*2015), sun bears (*Helarctos malayanus*) in Indonesia (Fredriksson, 2005) and American black bears in New Hampshire (Kilham, 2015; Smith *et al.,*2016). The trust and hands-on relationship between animals and humans that characterizes this soft release method enabled us to collect blood samples voluntarily from pandas in our program to better monitor their health status pre- and post-release.

Physiological research provides a useful tool in evaluating the health of the animal at individual and population levels in conservation (Homyack, 2010; Yang *et al*., 2018b). It reveals physiological processes influenced by the environment in which animals live (Ricklefs and Wikelski, 2002). Blood hematology and biochemistry levels, in particular, are a measure of the impact of environmental change on adaptation of animals (O’Connor *et al.,*1994; Cherry *et al.,*2009; Wintle *et al*., 2018).

Reference intervals (RIs) of blood parameters play a key role in laboratory diagnostic testing and clinical decision-making processes by forming a background ‘normal’ basis of comparison (Friedrichs *et al.,*2012). RIs are important diagnostic tools for assessing animal health not only of an individual but also at population levels (Yang *et al*., 2018b) and has important conservation applications. We hypothesized that by comparing RIs of blood from captive giant pandas to our release pandas, we could predict survivability of giant pandas in reintroduction programs as shown in other animals (Mathews *et al.,*2006; Graesli *et al.,*2014).

Establishing RIs for blood parameters in wild animals presents a challenge because capture stress or anesthesia during sampling can affect the resulting values (Casas-Díaz *et al.,*2015). In addition, sample size can be a problem. The International Federation of Clinical Chemistry and Laboratory Medicine (IFCC) recommends estimating RI on at least 120 subjects in human studies (Hansen *et al.,*2007). However, it is difficult to meet the requirement of 120 individuals for many endangered species because of small population sizes (Reiss *et al.,*2008; Lewbart *et al.,*2018; Priambada *et al.,*2021). Given the small and scattered populations of giant pandas in the wild and a globally distributed captive population of 673 animals it is very difficult to achieve that sample size. However, following Friedrichs *et al.* (2012) it is possible to determine RIs for rare animals with samples from fewer than 120 individuals (Yu *et al*., 2021).

Altitude plays an important role in the acclimatization of animals’ blood physiology and impacts their physiological status (Kiran *et al.,*2012). Due to the low atmospheric pressure, high altitude areas have a lower partial pressure of oxygen and lower ambient temperature. An animal that lives at high altitude develops special physiological adaptations in its blood physiology (Schmidt-Nielsen, 1975). For example, llamas (*Lama spp*.) that live at about 5000 m in the Andes of South America have a high hemoglobin affinity and take up oxygen at low atmospheric pressures (Schmidt-Nielsen, 1997). Oxygen dissociation curves (ODC) of high-altitude animals such as llama (*Lama glama*) and vicuna (*Lama vicugna*) show a leftward shift in the ODC compared to other mammals native to low altitudes resulting in increased ability for hemoglobin to retain or release oxygen (Hall *et al.,*1936; Schmidt-Nielsen, 1997). There may also be dynamic variations in the upregulation of 2,3-bisphosphoglycerate at altitude that shifts the Hb binding curve to the right and allows for a difference in O_2_ binding and release that maintains needed levels of O_2_ in the tissues for survivability at high altitude (Benesch and Benesch, 1967; Mulquiney *et al.,*1999; González-Cinca *et al.,*2004). Giant pandas inhabit montane environments; therefore, it is expected that they should have adaptations to high elevations reflected in their blood physiology.

In this study, we (1) established RIs for blood (hematological and biochemical) parameters of captive female giant pandas; (2) hypothesized that hematological and biochemical parameters of soft release giant pandas would differ from their captive controls; and (3) hypothesized that giant pandas would have different hematological and biochemical values when moved from low to high elevation.

This study is the first to compare RI for blood parameters of giant pandas and the first study to provide information on blood parameters of giant pandas living in semi-natural habitats. It creates a baseline for clinical care of captive giant pandas and post-release monitoring of giant pandas in the future.

## Materials and Methods

### Subjects

A total of 33 pandas were selected for the study, all of which were captive born at the Chengdu Research Base of Giant Panda Breeding (Panda Base) in Chengdu, Sichuan Province, China. Thirty pandas were maintained in captivity at the Panda Base in Chengdu, and three pandas were part of the soft-release program (Table 1). All the pandas were apparently healthy sub-adult and adult females that were part of a species propagation program. All pandas were routinely evaluated by staff veterinarians and received prophylactic medical care including regular treatment with anthelmintics as well as vaccinations for rabies and canine distemper viruses. Beyond prophylactic care, no pandas had received any medical pharmaceuticals (including anesthesia) for at least one month prior to blood sample collection for inclusion in this study. Since the translocation program for giant pandas was just recently started at the Panda Base, our study only focused on three giant pandas in the program that moved to large enclosures in nature reserves and stayed in the enclosures for more than 2 years. Pandas selected for the release program were transitioned into semi-wild enclosures at three locations: (1) Chengdu Research Base of Giant Panda Breeding Dujiangyan Wild Release Research Center (Panda Valley), (2) Liziping Nature Reserve and (3) Daxiangling Nature Reserve. After release into the enclosures, we monitored the pandas with GPS transmitters. This study was approved by the Institutional Animal Care and Use Committee of Chengdu Research Base of Giant Panda Breeding and Drexel University.

**Table 1:** Information on captive giant pandas at Chengdu Research Base of Giant Panda Breeding (Panda base) in Sichuan, China; and on release giant pandas at Panda Valley in Sichuan, China Liziping Nature Reserve in Sichuan, China and Daxiangling Nature Reserve in Sichuan, China.

Name	Group	ID	Sex	Birthdate	Age	Location
Run Jiu	Captive	1145	F	2018/7/31	4	Panda Base
Ni Na	Captive	1082	F	2017/7/20	5	Panda Base
Da Mei	Captive	1073	F	2017/6/27	6	Panda Base
Xing Fan	Captive	1033	F	2016/8/12	6	Panda Base
Fu lai	Captive	1014	F	2016/7/14	6	Panda Base
Qi Guo	Captive	1009	F	2016/7/1	7	Panda Base
Qi Yi	Captive	1008	F	2016/7/1	7	Panda Base
Yuan Man	Captive	1006	F	2016/6/29	7	Panda Base
Yuan Yue	Captive	1005	F	2016/6/29	7	Panda Base
Ji Lan	Captive	1004	F	2016/6/27	7	Panda Base
Ya Zhu	Captive	997	F	2016/6/20	7	Panda Base
Ni Da	Captive	965	F	2015/8/7	7	Panda Base
Fu Wa	Captive	948	F	2015/6/22	8	Panda Base
Ke Da	Captive	946	F	2015/6/22	8	Panda Base
Xing Yu	Captive	914	F	2014/6/30	9	Panda Base
Mei Huan	Captive	871	F	2013/7/15	9	Panda Base
Mei Lun	Captive	870	F	2013/7/15	9	Panda Base
Miao Miao	Captive	855	F	2012/9/4	10	Panda Base
Yuan Run	Captive	853	F	2012/8/25	10	Panda Base
Shuang Xin	Captive	818	F	2011/8/9	11	Panda Base
Ai Li	Captive	811	F	2011/7/24	11	Panda Base
Ya Yun	Captive	796	F	2010/8/10	12	Panda Base
Bei Chuan	Captive	765	F	2006	16	Panda Base
Zhi Zhi	Captive	763	F	2009/8/23	13	Panda Base
Ya Li	Captive	762	F	2009/7/19	13	Panda Base
Wen Li	Captive	761	F	2009/7/19	13	Panda Base
Qi Fu	Captive	709	F	2008/7/26	14	Panda Base
Xing Rong	Captive	680	F	2007/8/13	15	Panda Base
Ke Lin	Captive	678	F	2007/8/13	15	Panda Base
Ya Zai	Captive	637	F	2006/8/19	16	Panda Base
He Yu	Release	1029	F	2016/8/9	6	Panda Base,Panda Valley,Daxiangling Nature Reserve
Xing Chen	Release	1000	F	2016/6/21	7	Panda Base,Panda Valley,Daxiangling Nature Reserve
Qian Qian	Release	881	F	2013/8/6	9	Panda Base,Panda Valley,Liziping and Daxiangling Nature Reserve

### Study sites

#### Panda Base

**Figure 1:**
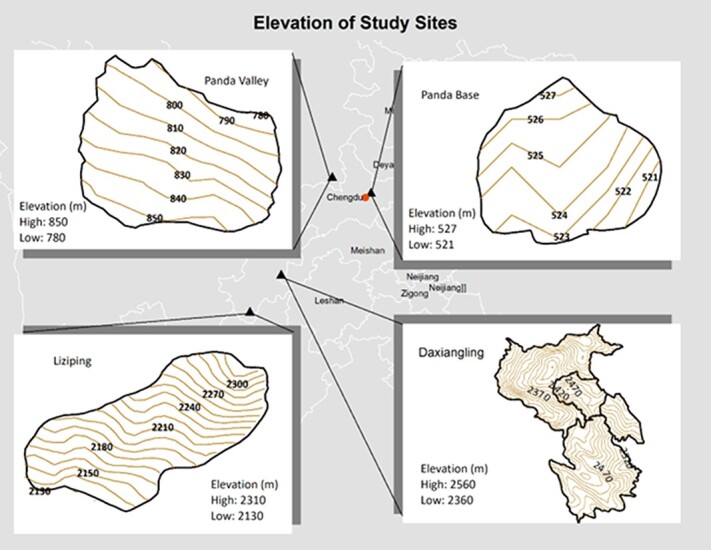
Maps of enclosures and elevations at the Chengdu Research Base of Giant Panda Breeding (Panda base) in Sichuan China Panda Valley in Sichuan China; Liziping Nature Reserve in Sichuan China and Daxiangling Nature Reserve in Sichuan China (maps are not to scale).

Panda Base was established as a nonprofit organization in 1987 as a rescue and breeding facility for giant pandas and a tourist location open to the general public. The main functions of the Panda Base were giant panda breeding, research, conservation, education, educational tourism and giant panda reintroduction. Captive pandas were maintained in enclosures with concrete interiors and natural outside areas with soil, grass and climbing structures, and were fed a diet of bamboo, bamboo shoots, apples and ‘panda cake’, a biscuit made of a mixture of grains with vitamins at Panda Base. The diet was consistent with established husbandry guidelines at Panda Base. Release pandas lived in a 0.77 ha natural enclosure and ate bamboo provided by the husbandry staff. The mean annual temperature was 16°C and the annual rainfall was 1000 mm. The altitude of the Panda Base was 524 m asl (Fig 1).

#### Panda Valley

Panda Valley, located in Dujiangyan, a city 60 km west of Chengdu, served as a semi-wild facility for and operated by Panda Base. The facility also included traditional enclosures for public viewing. We built a 3.8-hectare semi-wild learning enclosure for reintroduction pandas, which was approximately 815 m asl (Fig 1). The enclosure had natural vegetation and soil. The release pandas in this study spent their time outside in the learning enclosure and ate natural growing bamboo and provisioned bamboo brought into the enclosure to supplement the natural bamboo. The mean annual temperature was 15.2°C and the annual rainfall was 1200 mm.

#### Liziping nature reserve

Liziping Nature Reserve was located in the western part of Sichuan Province. The highest peak was 4551 m asl, and the elevation of the giant panda enclosure was 2220 m asl (Fig 1). The climate was humid, annual rainfall ranged from 800–1250 mm and the mean annual temperature was 11.7–14.4°C. The reintroduction enclosure was 23.1 ha in size and included natural vegetation and streams. Pandas ate naturally growing bamboo.

#### Daxiangling nature reserve

Daxiangling Nature Reserve was in the western part of the Sichuan Basin. The highest peak was 3553 m asl, and elevation ranged from 1500 to 3553 m asl (Xu *et al.,*2006). The climate was humid, annual rainfall was typically 1300–2000 mm and the mean annual temperature was 16°C. Below 1500 m elevation, the vegetation was mainly broad-leaved forest, at 1500–2500 m, it was mainly mixed forest, and above 2500 m asl, it was primarily coniferous forest (Zhao *et al.,*2017). The reintroduction giant pandas lived in enclosures of 13.3 and 49.4 ha at an elevation of 2456–2495 m asl (Fig 1). Pandas ate naturally growing bamboo.

### Blood collection and analysis

Traditional blood sampling methods, such as immobilization with medetomidine-tiletamine-zolazepam delivered by an anesthetic dart rifle, can increase an animal’s stress. Stress can increase the release of glucocorticoid hormones such as cortisol and corticosterone and decrease serum glucose, fat and protein (Reeder and Kramer, 2005). Additionally, the use of anesthetics regardless of the delivery method may also affect blood chemistry levels. For example, medetomidine has been verified to increase the blood glucose level in wild boar (*Sus scrofa*), red deer *(Cervus elaphus*) (Wolkers *et al.,*1994), black bear (Reeder and Kramer, 2005) and polar bear (*Ursus maritimus*) (Cattell *et al.,*1997; Cherry *et al.,*2009). Therefore, blood samples collected in a manner that induces stress and/or while anesthetized may reduce their comparability with non-stressed individuals and may not reflect their normal values.

**Figure 2:**
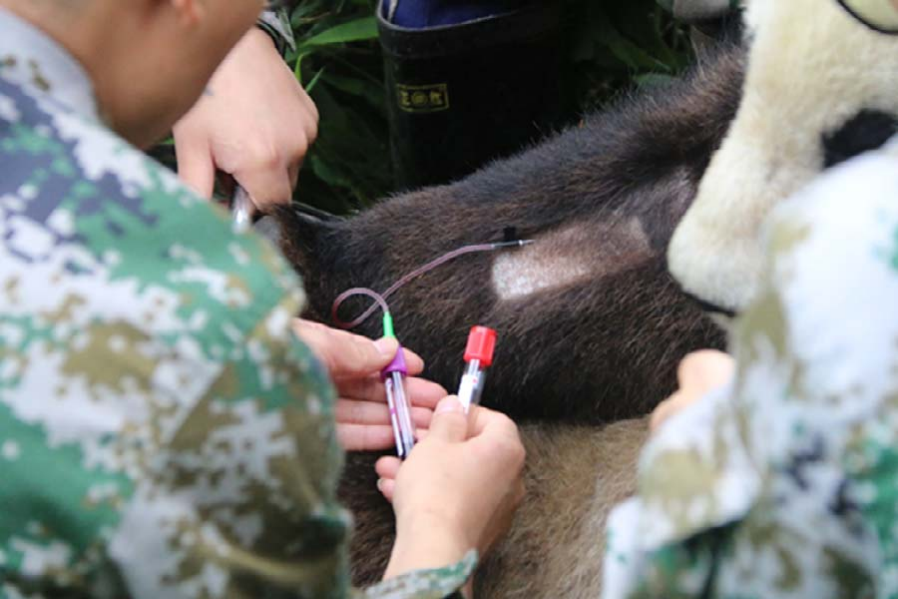
First author taking a blood sample from one of the soft release program giant pandas at Chengdu Research Base of Giant Panda Breeding at Daxiangling Nature Reserve in Sichuan China.

We collected blood samples from the giant pandas without anesthesia by training them to voluntarily extend their forelimb for cephalic vein venipuncture, thereby minimizing the effects of the sampling process (Fig 2). We fed up to three apples to the panda during the procedure as a treat so that it was rewarded and did not exhibit any stress. All pandas were between 1 and 16 years old. Most of the blood samples were collected in the morning between 9 am to 11 am. For the captive pandas, the blood was collected over ten years by veterinarians at Panda Base for health monitoring purposes using the voluntary blood collection method as part of their normal care procedures. Blood from the release pandas was collected by the authors or Panda Base veterinarians under the authors’ supervision. We located the free ranging released pandas in large enclosures using GPS transmitters. We obtained a blood sample from a cephalic vein using a 22 G butterfly catheter (B.D. Medical, Franklin Lakes, N.J., USA). We put the blood samples into a vacuum blood collection tube (B.D. Vacutainer, 5 ml, N.J., USA) treated with heparin to prevent coagulation (Graesli *et al.,*2014, Wintle *et al*., 2018). Blood samples were immediately placed unfrozen in a portable ice chest. There were 494 blood samples from 30 captive pandas and 68 blood samples from 3 release giant pandas. All blood samples were stored at 4°C (Yang *et al*., 2017) and sent to the blood test center in 416 Nuclear Industrial Hospital and other human hospitals in Sichuan Province, China, for analysis within 24 hours. Blood was analyzed with a Mindray 5 Differential Part Bc-5800 hematology analyzer (Mindray Technology Company, China) and an AU2700 biochemistry blood analyzer (Olympus, Japan).

### Statistical analysis

Data were collected on 36 different blood parameters, typically used as diagnostic measures in humans and animals. We eliminated derived values that were calculated from other reported parameters (e.g. BUN/creatinine ratio, albumin/globulin ratio, mean corpuscular hemoglobin concentration). We also eliminated differential white blood cell (WBC) counts that were done several different ways by machine and were not clearly comparable among samples. This left 26 parameters for analysis. We grouped these parameters into five different functional groups, potentially related to hypotheses about the pandas: (1) Electrolytes–serum concentrations of functionally and osmotically important ions present at millimolar concentrations (Na+, K+, Cl-, CO_2_ (mostly HCO3-), Ca++, Mg++), (2) nutrition or body condition related measures—energy substrates or indicators of protein balance (Albumin, Globulin, blood urea nitrogen [BUN], creatinine [CRE], glucose, cholesterol [CHO]), (3) Enzymes–serum concentrations of intracellular proteins used to monitor tissue inflammation or injury (alanine aminotransferase [AAT], aspartate aminotransferase [DAT], lactate dehydrogenase [LDH], alkaline phosphatase [ALP], creatine kinase [CK], Amylase), (4) miscellaneous chemical measures (not fitting the earlier groups (direct and indirect bilirubin [DBIL, IBIL], serum iron [Fe], uric acid) and (5) hematologic measures (white blood cell counts [WBC], hemoglobin concentration [Hb], hematocrit [Hct] and platelet count [Plt]). All of the enzymes and platelet counts were log-normally distributed and were log transformed for analysis of the comparisons between groups of pandas.

We determined the RI for the blood parameters of the captive giant pandas using the ASVCP reference guidelines as described in Friedrichs *et al.* (2012). We did not have the same number of samples for each parameter because of scattered missing values. First, we removed outliers in the data using Horn’s algorithm using Tukey’s interquartile fences to identify multiple outliers located at the upper and lower extremities. We eliminated between 51–65 samples out of 401–506 samples depending upon the parameter. We eliminated 1447 out of a total of 11 352 samples leaving 9905 samples in the data base. Then we calculated the RI using Reference Value Advisor freeware using the nonparametric method (Friedrichs *et al.,*2012).

To investigate whether blood measures differed among groups of pandas (captive and release), we employed both multivariate analyses, applied to functional groups of measures (above), and univariate analyses, applied to individual metrics. Multivariate analyses (PCA and MANOVA, executed in MATLAB (MATLAB, 2020) and R (R Core Team, 2020) could be applied to entire functional groups (e.g. the nutrition/condition group), but take a single blood sample as their unit of replication, rather than an individual under captive or release conditions. Thus, they are, to some extent, pseudoreplicated (Hurlbert, 1984). While multivariate analyses are useful in detecting and displaying differences among groups, the P values reported for differences among groups are likely to be undependable. Therefore, for significance testing, univariate statistics were run as repeated-measures analyses via the lmer function in the lme4 package in R (Bates *et al.,*2015). We used a Chi-square test to test for significance of location by comparing two models—one with the location included, and a reduced model without. The Chi-square statistic is lmer’s favored test parameter for the difference in log-likelihoods of the two models.

We compared captive vs reintroduction pandas (while they were still maintained at Panda Base). We also compared the effect of elevation and release (Panda base, Panda Valley, Daxiangling and Liziping) on the release pandas. We accepted P < 0.01 for statistical significance.

## Results

We obtained 562 blood samples from 33 individual giant pandas (Table 1). We analyzed those samples for the 26 different parameters described in the Methods (defined in Table 2). After eliminating outliers, we calculated RI for each parameter for the 30 captive giant pandas (Table 3). Descriptive statistics summarized the observations for release pandas (Table 4).

**Table 2:** Case processing summary for blood samples from giant pandas at Chengdu Research Base of Giant Panda Breeding (Panda base) in Sichuan, China.

Case Processing Summary						
Classification	Parameters			Sample Size		
	Abbreviation	English term	Unit	Captive	Release	Total
Hematology	WBC	White blood cell	×10^9^/L	412	64	476
Hematology	Hb	Hemoglobin	g/L	421	64	485
Hematology	Hct	Hematocrit	%	419	64	483
Hematology	Plt	Blood platelet	×10^9^/L	422	64	486
Miscellaneous chemistries	DBIL	Direct Bilirubin	umol/L	384	65	449
Miscellaneous chemistries	IBIL	Indirect Bilirubin	umol/L	405	64	469
Miscellaneous chemistries	Uric Acid	Uric acid	umol/L	437	64	501
Miscellaneous chemistries	Fe	Iron	umol/L	383	52	435
Enzymes	DAT	Aspartate aminotransferase	U/L	390	65	455
Enzymes	AAT	Alanine aminotransferase	U/L	387	65	452
Enzymes	ALP	Alkaline phosphatase	U/L	413	64	477
Enzymes	LDH	Lactate dehydrogenase	U/L	403	63	466
Enzymes	Amylase	Amylase	U/L	421	61	482
Enzymes	CK	Creatinine kinase	umol/L	404	64	468
Nutrition	BUN	Blood urea nitrogen	mmol/L	437	62	499
Nutrition	CRE	Creatinine	U/L	420	65	485
Nutrition	Globulin	Globulin	g/L	430	65	495
Nutrition	Albumin	Albumin	g/L	437	65	502
Nutrition	Glucose	Glucose	mmol/L	420	65	485
Nutrition	CHO	Cholesterol	mmol/L	433	64	497
Electrolytes	CO_2_	Carbon dioxide	mmol/L	418	65	483
Electrolytes	Ca	Calcium	mmol/L	351	51	402
Electrolytes	K	Potassium	mmol/L	435	63	498
Electrolytes	Na	Sodium	mmol/L	433	63	496
Electrolytes	Cl	Chlorine	mmol/L	425	63	488
Electrolytes	Mg	Magnesium	mmol/L	421	65	486

Samples were from captive giant pandas at two facilities of Panda base and from giant pandas in a reintroduction program at those facilities and in two nature reserves.

**Table 3:** RIs calculated with nonparametric methods for 26 parameters for blood samples from giant pandas at Chengdu Research Base of Giant Panda Breeding (Panda base) in Sichuan, China.

Group	Parameter	*n*	Mean	SD	RI	90% CI for lower limit		90% CI for upper limit	
						Min	Max	Min	Max
Electrolytes	Ca	351	1.10	0.17	0.75–1.47	0.72	0.79	1.42	1.51
	Cl	425	97.34	3.47	90.50–104.44	90.00	90.90	103.77	105.40
	CO_2_	418	20.60	2.57	15.90–25.87	14.50	16.34	25.65	26.15
	K	435	4.94	0.62	3.93–6.15	3.83	4.01	6.03	6.29
	Mg	421	0.99	0.10	0.82–1.20	0.78	0.83	1.17	1.23
	Na	433	129.74	3.16	124.20–136.00	123.35	124.87	135.86	137.00
Enzymes	ALP	413	175.99	76.97	75.35–360.65	73.51	80.35	343.60	379.00
	AAT	387	113.95	56.63	43.70–268.80	41.66	47.00	259.74	278.00
	Amylase	421	811.29	385.28	205.75–1580.95	184.40	232.80	1524.00	1659.11
	DAT	390	62.03	28.33	28.00–141.00	26.00	30.00	137.00	146.00
	CK	404	228.53	111.12	89.00–508.38	84.25	93.00	471.00	530.00
	LDH	403	902.35	247.65	510.20–1478.10	460.00	550.00	1414.20	1524.58
Hematology	Hct	419	32.83	3.43	26.20–40.05	25.55	26.75	39.10	40.25
	Hb	421	117.05	12.37	92.00–143.00	89.00	94.00	140.00	145.00
	Plt	422	613.41	166.94	369.73–1004.78	335.00	394.97	957.16	1032.00
	WBC	412	8.32	1.74	4.81–11.93	4.60	5.24	11.77	12.24
Miscellaneous chemistries	DBIL	384	0.48	0.26	0.09–1.10	0.07	0.10	1.01	1.24
	Fe	383	32.82	6.90	19.10–47.46	18.18	20.32	46.37	49.28
	IBIL	405	0.42	0.31	0.03–1.20	0.02	0.03	1.09	1.30
	Uric Acid	437	61.65	27.67	25.39–123.53	23.26	28.19	121.00	127.04
Nutrition	Albumin	437	34.90	2.68	29.49–39.82	28.60	29.99	39.13	40.70
	BUN	437	6.51	4.01	1.37–15.51	1.29	1.67	14.63	16.55
	CRE	420	85.88	24.51	40.77–137.66	39.33	45.80	132.40	151.20
	Globulin	430	28.46	4.20	19.81–36.80	18.70	20.48	35.50	37.57
	Glucose	420	3.65	0.97	1.83–5.68	1.71	2.03	5.51	5.97
	CHO	433	5.57	1.09	3.57–8.01	3.46	3.77	7.67	8.28

Samples from Panda Base were from captive giant pandas at two facilities of Panda base.

**Table 4:** Values for blood samples from giant pandas in a reintroduction program in two nature reserves and the Chengdu Research Base of Giant Panda Breeding (Panda Base) in Sichuan, China.

Classification	Parameter	Release		
		*n*	Mean	SD
Hematology	WBC	64	8.5	2.05
Hematology	Hb	64	125.06	16.56
Hematology	Hct	64	35.87	4.95
Hematology	Plt	64	488.88	108.17
Miscellaneous chemistries	DBIL	65	0.71	0.66
Miscellaneous chemistries	IBIL	64	0.57	0.7
Miscellaneous chemistries	Uric Acid	64	49.48	22.31
Miscellaneous chemistries	Fe	52	35.13	9.88
Enzymes	DAT	65	65.49	36.35
Enzymes	AAT	65	111.88	74.81
Enzymes	ALP	64	276.6	155.98
Enzymes	LDH	63	921.55	322.95
Enzymes	Amylase	61	548.76	161.45
Enzymes	CK	64	387.97	267.34
Nutrition and condition	BUN	62	9.07	4.38
Nutrition and condition	CRE	65	78.92	20.28
Nutrition and condition	Globulin	65	30.2	3.63
Nutrition and condition	Albumin	65	35.91	3.71
Nutrition and condition	Glucose	65	4.03	0.96
Nutrition and condition	CHO	64	6.41	1.71
Electrolytes	CO_2_	65	18.35	3.23
Electrolytes	Ca	51	1.04	0.22
Electrolytes	K	63	5.08	0.71
Electrolytes	Na	63	124.81	4.04
Electrolytes	Cl	63	94.98	4.14
Electrolytes	Mg	65	1.13	0.16

**Figure 3:**
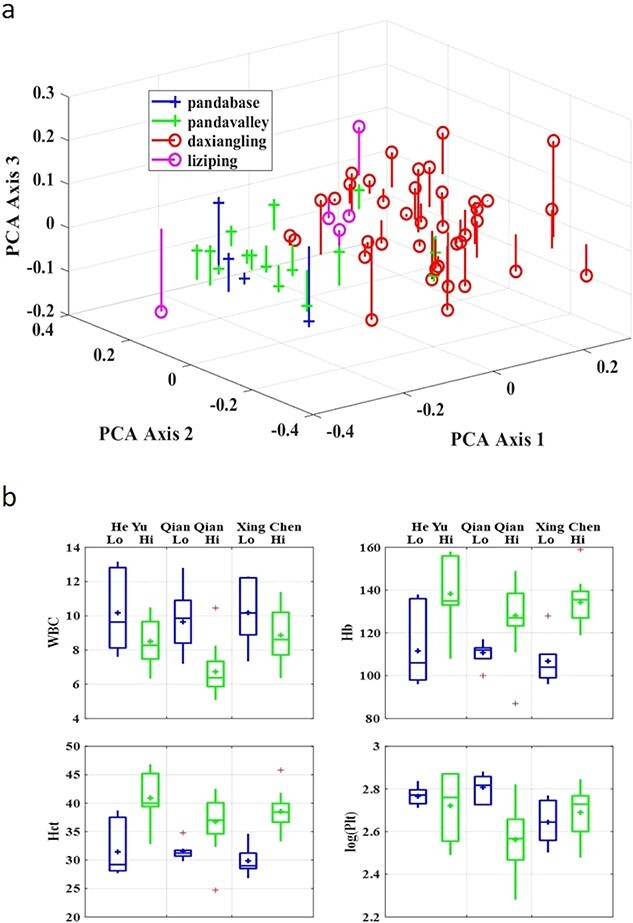
Hematology PCA and MANOVA results of release pandas at low elevation (Chengdu Research Base of Giant Panda Breeding and Panda Valley in Sichuan China), and high elevation (Liziping Nature Reserve in Sichuan China, and Daxiangling Nature Reserve in Sichuan China). a. Plot of individual samples vs first three axis scores for PCA run on hematologic values, with drop lines to help locate the points in the x, y plane (axis 1 score and axis 2 score are the same as the point and axis 3 score = 0). The PCA plot splays the sample points out in space along the 1st three PCA axes to show the groupings. (b) Box plot of univariate results from the MANOVA analysis of four parameters for each individual at low (524–815 m asl) and high (2220–3552 m asl) elevations.

We tested differences in blood parameters of release pandas at different elevations. The release giant pandas had significantly different hematology at high elevation than at low elevation (MANOVA, Hotelling-Lawley, df = 4,59, p < 0.001). The release giant pandas had higher Hb and Hct values but lower WBC at high elevation than at low elevation (Table 5 and Fig 3). The PCA plot Fig 3a) visually presented the fairly strong separation between the low altitude sites (Panda Base and Panda Valley) and the high altitude site (Daxiangling). The PCA plots were done in three axes because the Scree plots suggested the need to retain three axes. There were insufficient samples from Liziping for an adequate comparison.

**Table 5:** Significantly different blood parameters of release pandas at low (524–815 m asl) and high (2220–3552 m asl) elevation sites and of release pandas and captive pandas at low elevation (when p < target is yes). Blood samples from giant pandas at low elevation were from the Chengdu Research Base of Giant Panda Breeding (Panda base) and Panda Valley, and high elevation sites included Liziping Nature Reserve (Shimian County) and Daxiangling Nature Reserve (Yingjing County) in Sichuan, China.

Group	Parameter	Different elevation in release pandas					Captive vs release at low elevation				
		High	Low	ChiSq	*P*	*P* < target	Captive	Release	ChiSq	*P*	*P* < target
Nutrition/condition	Albumin	37.16	33.08	19.7	9.08E-06	Yes	34.36	33.03	1.6	2.07E-01	
	Globulin	31.35	27.6	17.1	3.60E-05	Yes	28.91	27.59	0.87	3.52E-01	
	BUN	9.75	7.04	6.2	1.30E-02		6.13	7	0.5	4.80E-01	
	Cre	86.3	62.34	23.4	1.33E-06	Yes	87.26	62.68	4.5	3.39E-02	
	Glucose	3.99	4.12	0.3	6.21E-01		3.85	4.12	0.75	3.87E-01	
	Cho	6.29	7.07	4.7	2.94E-02		5.9	7.05	4.05	4.40E-02	
Electrolytes	Na	124.29	124.71	0.3	5.98E-01		129.14	124.78	12.8	3.50E-04	Yes
	K	5.12	5	0.4	5.33E-01		4.91	5	0.3	6.01E-01	
	Cl	94.65	94.98	0.1	7.80E-01		97.65	95	6.3	1.24E-02	
	CO_2_	18.19	18.75	0.5	4.75E-01		20.39	18.81	3.9	4.75E-02	
	Ca	1.048	1.031	0.1	7.89E-01		1.107	1.03	2.3	1.28E-01	
	Mg	1.196	1.003	22.4	2.20E-06	Yes	1.004	1.002	0.003	9.58E-01	
Enzyme (log[])	DAT	1.861	1.525	49.6	1.89E-12	Yes	1.77	3.8	7.1	7.92E-03	
	AAT	2.029	1.847	11.7	6.23E-04	Yes	2.03	1.85	4.2	4.05E-02	
	LDH	2.945	2.847	13.5	2.33E-04	Yes	3.01	2.85	3.6	5.92E-02	
	Alp	2.322	2.594	28.9	7.64E-08	Yes	2.3	2.59	7.6	5.84E-03	
	Ck	2.595	2.3	21.8	3.07E-06	Yes	2.35	2.3	0.7	3.99E-01	
	Aamylase	2.766	2.592	33.9	5.90E-09	Yes	2.77	2.59	2.2	1.38E-01	
Miscellaneous chemistries	Dbil	0.79	0.539	2.1	1.52E-01		0.8	0.54	1.5	2.25E-01	
	Ibil	0.674	0.34	3.2	7.41E-02		0.57	0.34	2.1	1.52E-01	
	Fe	35.37	35.62	0.01	9.04E-01		34.17	35.62	0.3	5.75E-01	
	Uric Acid	51.19	45.71	0.8	3.57E-01		57.39	45.45	1.9	1.64E-01	
Hematology	Wbc	7.95	10.02	19.7	1.26E-05	Yes	8.55	9.99	4.7	3.07E-02	
	Hb	132.25	109.86	31.6	1.94E-08	Yes	115.97	109.77	1.9	1.69E-01	
	Hct	38.29	31.01	37.7	8.42E-10	Yes	32.43	30.99	1.4	2.30E-01	
	log(Plt)	2.64	2.74	8.1	4.33E-03		2.77	2.74	0.5	4.93E-01	

**Figure 4:**
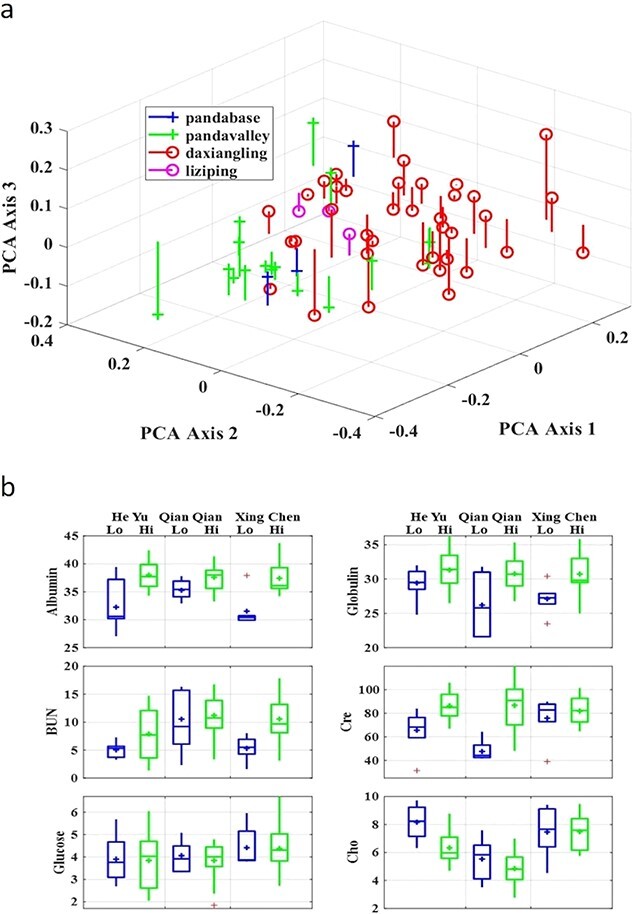
Nutrition and condition PCA and MANOVA results of release pandas at low elevation (Chengdu Research Base of Giant Panda Breeding and Panda Valley in Sichuan China), and high elevation (Liziping Nature Reserve in Sichuan China, and Daxiangling Nature Reserve in Sichuan China). (a) Plot of individual samples vs first three axis scores for PCA run on nutrition and condition values, with drop lines to help locate the points in the x, y plane (axis 1 score and axis 2 score are the same as the point and axis 3 score = 0). The PCA plot splays the sample points out in space along the 1st three PCA axes to show the groupings. (b) Box plot of univariate results from the MANOVA analysis of six parameters for each individual at low (524–815 m asl) and high (2220–3552 m asl) elevations.

For the miscellaneous chemistry parameters, we did not find a significant difference between high elevation and low elevation pandas (Table 5 and Fig 4). There was a significant difference in the enzyme parameters between high and low elevation pandas (MANOVA, Hotelling-Lawley, df = 6,53, p < 0.001). The PCA plot visualized the differences between low altitude and high altitude sites and also used three axes (Fig 4a). The release giant pandas had higher DAT, AAT, CK and Amylase values but lower LDH and ALP values at high elevation than at low elevation (Table 5). For the nutrition and condition parameters, the release giant pandas had higher values of Albumin, Globulin and CRE at high elevation than at low elevation (MANOVA, Hotelling-Lawley, df = 6,54, *P* < 0.001, Table 5). We did not find a significant difference in electrolyte parameters between different elevations except for Mg. The Mg value was significantly higher at high elevation compared to low elevation (MANOVA, Hotelling-Lawley, df = 6,44, *P* < 0.001).

We also compared all the parameters between the release pandas and captive pandas at Panda Base and Panda Valley. Only one parameter was significantly different between the two groups: the release group had a significantly higher Na value than the captive group (Chi Square, *P* = 3.50E-04, Table 5).

## Discussion

These are the first RI values determined for giant pandas and they can serve as a basis for further establishing baseline reference ranges for both captive giant pandas and those released into the wild in reintroduction programs. Previous studies did not determine RIs but did report means and SD. In general, means and SD of blood parameters in our study were similar to those in previous studies of captive giant pandas (Table 6) (Wang *et al.,*1998; Li *et al*., 1999; Li *et al*., 2012; Luo *et al.,*2017; Yu *et al.,*2019; Deng *et al.,*2020; Zeng *et al.,*2021). All our giant pandas were females. A 1998 study reported 26 biochemistry values from 18 giant pandas from the Chengdu Zoo and Panda Base. There were no significant differences between males and females in any parameters they assessed, except for Fe, which was significantly different between cubs (< 2 years old) and adults (Wang *et al.,*1998). Another study on 47 physiological and biochemical values of 14 captive giant pandas from Panda Base and Chengdu Zoo only found differences in ALP, neutrophil granulocyte and lymphocyte between subadult and adult giant pandas (Li *et al*., 1999). A study comparing 20 physiological blood parameters on 120 captive giant pandas from the China Conservation and Research Center for Giant Pandas reported significant differences in red blood cell count (RBC) and WBC between males and females; lymphocyte and mean hemoglobin concentrations (MHb) between old age and adults; RBC and MHb between old age and subadults; WBC between adults and cubs; and WBC and Hb between cubs and subadults (Li *et al*., 2012). Hematologic and biochemical parameters for 114 captive giant pandas from Panda Base determined using non-stress (voluntary blood draw) sampling methods in 2017 (Luo *et al.,*2017) varied more by sex for adult pandas than when comparing between different ages. When examining data from past studies in comparison to our RIs we believe all values measured for giant pandas to date have been in normal physiological ranges. Variations between studies were likely caused by individual and environmental variations at the different sites. The RIs that we report are limited because only females were evaluated with pandas in all reproductive stages represented. Additional studies are needed to more completely understand the RIs of giant pandas in captivity and the wild especially in regard to factors that are likely to affect blood parameters such as season, reproductive status, diet, natural endo- & ecto-parasitism, prophylactic medical therapies and captivity related stress.

**Table 6:** Comparison of parameter statistics for blood samples from giant pandas at the Chengdu Research Base of Giant Panda Breeding (CRB) in Sichuan China in other studies.

Study	Age group	Statistics	Nutrition Condition						Enzyme						
			Albumin	Globulin	BUN	CRE	Glucose	CHO	DAT	AAT	LDH	ALP	CK	Amylase	
Guanghan Li et at.,1991 18 pandas	NA	n	7	10	17	18	13	9	9	Na	16	3	Na	14	
		mean	26.1	32.9	5.15	110.7	4.8	8.01	31	Na	522	335	Na	466.9	
		SD	2	4	0.63	15.1	0.4	1.4	9	Na	209	87	Na	130.1	
Wang et al., 1998 18 pandas	NA	n	33	33	26	26	21	NA	28	Na	24	24	Na	21	
		mean	34.88	30.21	4.74	103.61	4.3	NA	33.4	Na	413.04	121.29	Na	547.96	
		SD	4.45	7.51	1.36	28.12	1.41	NA	17.87	Na	237.46	28.29	Na	114.44	
Li Luo et al., 2017 114 pandas	Adult	n	63	62	63	62	61	62	60	62	63	62	55	61	
		mean	36.35	29.19	8.36	109.7	3.32	5.17	88.65	145.44	856.12	150.53	397.32	1000.3	
		SD	1.72	2.41	2.23	19.48	0.48	0.83	24.88	50.37	321.75	30.19	174.08	202.5	
	Sub-adult	n	63	53	60	63	63	63	62	61	63	63	30	63	
		mean	35.89	27.95	8.3	98.21	3.18	5.44	67.5	113.69	1024.19	198.85	363.34	768.66	
		SD	2.01	3.15	2.88	24.93	0.63	1.09	15.05	31.64	372.14	59.58	138.53	187.48	
	Cub	n	48	48	48	44	48	48	48	48	48	48	18	47	
		mean	34.78	27.67	6.66	71.68	3.49	6.13	48.14	94.15	1100.75	304.66	321.26	545.69	
		SD	2.49	4.07	2.76	13.41	0.94	0.83	12.06	24.73	410.69	84.74	83.03	174.77	
Changmeng Yu et al., 2019 9 pandas	Aged	n	6	6	6	6	6	6	6	6	6	6	6	6	
		mean	31.18	39.48	4.42	82.11	3.02	3.68	47.2	83.7	3337.07	211.2	135.24	Na	
		SD	1.99	4.95	1.08	10.92	0.57	1.39	13.36	24.13	1259.79	32.31	82.44	Na	
	Adult	n	15	15	15	15	15	15	15	15	15	15	15	15	
		mean	31.68	33.47	5.17	85.64	3.66	5.38	53.81	85.82	2257.51	210.44	225.03	Na	
		SD	3.33	3.08	1.44	19.94	1.09	0.92	15.49	22.82	1023.89	59.64	192.94	Na	
	Infant	n	6	6	6	6	6	6	6	6	6	6	6	6	
		mean	32.52	30.82	4.92	74.39	4.14	4.9	37.33	115.57	2053.6	504.83	149.7	Na	
		SD	1.2	2.44	1.24	7.08	0.42	0.35	8.42	12.37	172.22	214.19	52.46	Na	
Linhua Deng et al., 2020 18 giant pandas	Aged	n	7	7	7	7	7	7	7	7	7	7	7	7	
		mean	30.56	35.49	4.77	118.25	3.82	5.58	71.93	81.76	623.11	117.07	Na	Na	
		SD	2.41	4.18	1.55	35.06	0.73	1.79	13.34	19.84	320.28	38.6	Na	Na	
	Adult	n	4	4	4	4	4	4	4	4	4	4	4	4	
		mean	30.92	33.38	4.57	102.22	4.23	4.48	72.19	120.29	525	142.88	Na	Na	
		SD	2.44	4.64	1.79	18.31	0.91	0.82	13.96	47.45	312.08	49.32	Na	Na	

Continued

**Table 6:** Continued

Study	Age group	Statistics	Nutrition Condition						Enzyme						
			Albumin	Globulin	BUN	CRE	Glucose	CHO	DAT	AAT	LDH	ALP	CK	Amylase	
	Sub-Adult	n	7	7	7	7	7	7	7	7	7	7	7	7	
		mean	33.47	29.68	4.85	104.06	4.57	5.56	58.39	123.24	552.96	200.5	Na	Na	
		sd	1.88	3.14	1.61	23.91	0.67	0.94	11	35.89	227	58.77	Na	Na	
Min Zeng et al., 2021 73 giant pandas	Old	n	8	8	8	8	8	8	8	8	8	8	8	8	
		mean	33.37	32.08	NA	NA	3.24	4.28	59.85	41.75	Na	130.15	Na	Na	
		SD	3.92	5.89	NA	NA	1.58	1.71	21.46	20.33	Na	38.89	Na	Na	
	Adult	n	35	35	35	35	35	35	35	35	35	35	35	35	
		mean	34.55	29.79	NA	NA	3.28	3.85	63.31	60.25	Na	117.51	Na	Na	
		SD	5.25	4.84	NA	NA	1.16	0.72	19.04	23.95	Na	50.16	Na	Na	
	Young	n	30	30	30	30	30	30	30	30	30	30	30	30	
		mean	35.35	24.22	NA	NA	2.81	4.56	57.03	75.67	Na	201.57	Na	Na	
		SD	7.28	2.48	NA	NA	1.15	1.19	24.26	29.29	Na	76.42	Na	Na	
**This study**	**Captive**	**n**	437	430	437	420	420	433	390	387	403	413	404	421	
**33 pandas**		**mean**	34.9	28.46	6.51	85.88	3.65	5.57	62.03	113.95	902.35	175.99	228.53	811.29	
		SD	2.68	4.2	4.01	24.51	0.97	1.09	28.33	56.63	247.65	76.97	111.12	385.28	
	**Release**	**n**	65	65	62	65	65	64	65	65	63	64	64	61	
		**mean**	35.91	30.2	9.07	78.92	4.03	6.41	65.49	111.88	921.55	276.6	387.97	548.76	
		SD	3.71	3.63	4.38	20.28	0.96	1.71	36.35	74.81	322.95	155.98	267.34	161.45	
Study	Age group	Statistics	Electrolytes					Miscellaneous chemistries				Hematology			
			Na	K	Cl	CO2	Ca	DBIL	IBIL	Fe	Uric Acid	Wbc	Hb	Hct	Plt
Guanghan Li et at., 1991 18 pandas	NA	n	10	19	15	14	9	Na	Na	10	Na	15	11	18	8
		mean	133.6	4.63	100.2	21.8	2.5	Na	Na	34.68	Na	8	131	0.08	418
		sd	3.8	0.82	2.5	1.6	0.1	Na	Na	12.25	Na	1.9	15	0.33	79
Wang et al., 1998 18 pandas	NA	n	32	30	31	25	30	Na	Na	24	Na	16	17	8	Na
		mean	132.63	4.62	102.26	18.88	2.37	Na	Na	17.57	Na	9.8	131.71	42.05	Na
		sd	8.24	0.6	6.26	2.41	0.29	Na	Na	7.67	Na	2.67	28.05	4.54	Na
Li Luo et al., 2017 114 pandas	Adult	n	63	63	63	62	63	62	63	62	63	62	63	62	63
		mean	129.98	5.02	97.74	20.43	2.22	0.52	0.59	35.19	74.96	8.11	126.57	34.87	552.79
		sd	1.63	0.32	1.57	1.23	0.1	0.19	0.3	3.43	16.94	1.19	11.55	2.66	106.55
	Sub-adult	n	63	63	63	63	63	59	62	63	63	63	63	63	63
		mean	129.59	5.13	96.91	20	2.39	0.41	0.65	32.43	82.63	8.18	115.39	32.16	593.84
		sd	2.43	0.37	2.76	1.56	0.18	0.22	0.31	6.77	19.41	1.73	10.98	2.8	121.53

Continued

**Table 6:** Continued

Study	Age group	Statistics	Nutrition Condition						Enzyme						
			Albumin	Globulin	BUN	CRE	Glucose	CHO	DAT	AAT	LDH	ALP	CK	Amylase	
	Cub	n	48	47	48	47	46	46	47	47	48	50	50	49	50
		mean	131.01	5.33	98.91	19.19	2.58	0.31	0.52	33.14	83.42	9.57	111.78	31.42	693.84
		sd	2.39	0.48	3.11	1.82	0.15	0.17	0.38	7.06	33.93	3.07	9.66	2.45	191.91
Changmeng Yu et al., 2019 9 pandas	Aged	n	6	6	6	6	6	6	6	6	6	6	6	6	6
		mean	125.17	4.8	92.68	22.77	1.97	1.77	0.71	Na	44.83	12.68	123.67	34.05	521.67
		sd	2.34	0.44	2.63	3.77	0.11	0.51	0.87	Na	9.57	2.89	8.77	2.97	73.79
	Adult	n	15	15	15	15	15	15	15	15	15	15	15	15	15
		mean	124.6	4.9	94.77	21.92	2.2	1.84	0.53	Na	43.23	7.32	125.6	33.75	559.2
		sd	2.42	0.4	3.71	2.54	0.09	0.42	0.75	Na	8.17	1.92	11.93	2.98	145.25
	Infant	n	6	6	6	6	6	6	6	6	6	6	6	6	6
		mean	125.17	4.57	95.48	20.37	2.31	1.79	0.73	Na	47.01	7.49	125.5	34.05	536.17
		sd	2.03	0.44	1.15	1.72	0.07	0.47	0.89	Na	3.59	0.53	6.75	2.49	91.17
Linhua Deng et al., 2020 18 giant pandas	Aged	n	7	7	7	7	7	7	7	7	7	7	7	7	7
		mean	124.04	5.03	94.03	Na	59.7	0.32	1.5	432.56	33.64	6.27	116.32	30.64	310.36
		sd	3.18	0.46	2.73	Na	6.03	0.25	0.64	39.3	14.96	1.37	9.76	2.79	107.53
	Adult	n	4	4	4	4	4	4	4	4	4	4	4	4	4
		mean	125.38	5.05	96.85	Na	58.33	0.45	1.36	472.28	41.06	7.28	129	33.66	360.06
		sd	1.7	0.73	2	Na	4.6	0.33	0.96	50.35	21.8	1.25	12.64	3.41	115.24
	Sub-Adult	n	7	7	7	7	7	7	7	7	7	7	7	7	7
		mean	125.73	5.05	97.18	Na	64.94	0.28	1.36	437.96	31.07	6.02	118.82	31.43	450.43
		sd	2.46	0.33	3.38	Na	4.91	0.13	0.55	39.88	16.05	0.95	8.6	3.23	77.08
Min Zeng et al., 2021 73 giant pandas	Old	n	8	8	8	8	8	8	8	8	8	8	8	8	8
		mean	Na	Na	Na	Na	2.04	0.53	0.42	28.63	55	6.32	121.32	33.41	316.5
		sd	Na	Na	Na	Na	0.02	0.06	0.03	10.99	20.81	2.04	15.33	5.61	59.59
	Adult	n	35	35	35	35	35	35	35	35	35	35	35	35	35
		mean	Na	Na	Na	Na	2.16	0.73	0.48	30.9	56.25	8.85	125.15	35.16	391.93
		sd	Na	Na	Na	Na	0.19	0.3	0.05	5.29	14.41	2.22	18.9	6.19	150.51
	Young	n	30	30	30	30	30	30	30	30	30	30	30	30	30
		mean	Na	Na	Na	Na	1.92	0.72	0.64	22.18	65.77	7.22	124.02	35.69	451
		sd	Na	Na	Na	Na	0.42	0.23	0.17	15.08	17.41	1.76	15.69	5.52	103.8
**This study**	**Captive**	***n***	433	435	425	418	351	384	405	383	437	412	421	419	422
**33 pandas**		**mean**	129.74	4.94	97.34	20.6	1.1	0.48	0.42	32.82	61.65	8.32	117.05	32.83	613.41
		**sd**	3.16	0.62	3.47	2.57	0.17	0.26	0.31	6.9	27.67	1.74	12.37	3.43	166.94

Continued

**Table 6:** Continued

Study	Age group	Statistics	Nutrition Condition						Enzyme						
			Albumin	Globulin	BUN	CRE	Glucose	CHO	DAT	AAT	LDH	ALP	CK	Amylase	
	**Release**	***n***	63	63	63	65	51	65	64	52	64	64	64	64	64
		**mean**	124.81	5.08	94.98	18.35	1.04	0.71	0.57	35.13	49.48	8.5	125.06	35.87	488.88
		**sd**	4.04	0.71	4.14	3.23	0.22	0.66	0.7	9.88	22.31	2.05	16.56	4.95	108.17

Samples from CRB were from captive giant pandas at two facilities of CRB, and from giant pandas in a reintroduction program at those facilities and in two nature reserves. Samples from other studies were from captive giant pandas.

There was a significant difference in WBC in release giant pandas before (in captivity) vs after release (with attendant changes in altitude). The WBC count was lower in the pandas after release. Although the differential was not analyzed, in the absence of observable clinical disease over the time period for blood draws, the overall lower WBC count is likely an indicator of reduced source of stress or inflammation. There were also differences in Albumin, Globulin, CRE and Mg as well as enzymes suggesting changes due to acclimation to high elevation (Hall *et al.,*1936; Hammond *et al.,*1999, 2001). A higher Fe content and lower unsaturated iron-binding capacity in captive brown bears than in wild bears were attributed to licking iron bars of cages (Huber *et al.,*1997). There was no difference in Fe content between captive and release giant pandas in our study. In Andean bears (*Tremarctos ornatus*), higher serum glucose and monocyte levels, but lower mean cellular Hb concentrations occurred in free-ranging individuals than in captive individuals (Castellanos *et al.,*2010). In our giant pandas, glucose and Hb levels were similar between captive and release animals when at low altitude. We are not aware of any other studies that compare the blood parameters of captive vs free-ranging giant pandas or bears. Therefore, additional studies are needed to more completely define the differences in blood parameters between released bears and their captive controls.

As with most free-roaming wild animal species, there are no published studies on the blood parameters of free-roaming giant pandas. Our study explored the acclimatization to altitude in giant pandas in large enclosures in nature reserves. We found our release program pandas had higher Hb, and Hct after moving to Daxiangling Nature Reserve (Table 6, Fig 3). Many studies indicate that animals that live at high elevations face an additional physiological challenge because of lower oxygen availability and low ambient temperature (Lu *et al.,*2015). Exercise can increase sympathetic activity and thus increases Hct (Wickler and Anderson, 2000). Red blood cell number increases significantly in horses when taken from low elevation (225 m) to high elevation (3800 m) (Wickler and Anderson, 2000). Lizards (*Genus Phrynocephalus*) that live at high elevation have higher oxygen-carrying capacity due to increased RBC, Hb and Hct (Lu *et al.,*2015). Therefore, we conclude that differences observed in Hb and Hct in giant pandas are reflective of acclimation to living at a high altitude.

Blood parameters have been reported in several bear species, including the American black bear (*Ursus americanus*) (Matula *et al.,*1980; Nelson *et al.,*1984; Schroeder, 1987; Karen and David, 1992; Hellgren *et al.,*1993; LeBlanc *et al.,*2001; Lohuis *et al.,*2005), brown bear (*Ursus arctos*) (Robert, 1985; Hissa *et al.,*1994; Huber *et al.,*1997; Graesli *et al.,*2014; Græsli *et al.*, 2015), polar bear (*Ursus maritimus*) (Derocher *et al.,*1990; Tryland *et al.,*2002; Sandala *et al.,*2004; Thiemann *et al.,*2008; Bytingsvik *et al.,*2012) Andean bear (*Tremarctos ornatus*) ([Bibr ref8][Bibr ref8]; Castellanos *et al.,*2013), sloth bear (*Melursus ursinus*) (Veeraselvam *et al.,*2014, 2018) and Malayan sun bear (*Helarctos malayanus*) (Azlan *et al.,*2011). The specific parameters assessed vary between studies based on their respective research focus. Most studies aimed to evaluate animal health status, hibernation adaptation and the bears’ basic physiology in free-ranging individuals (Graesli *et al.,*2014). It is surprising that there were no major differences in blood parameters between these bear species and the giant pandas evaluated in our study. On the one hand giant pandas are bears. However, we would expect that biochemical blood parameters might differ due to differences in diet. Castellanos *et al.* (2010) reported that the Andean bear feeding on a highly fibrous diet had 4.77 mmol/l blood urea nitrogen, which was lower than that of the giant panda in our study. However, alkaline phosphatase levels were similar to those of the giant panda. Polar bears are obligate carnivores and live on high protein and fat based diets. Giant pandas are primarily vegetarians and eat mostly bamboo. Bamboo shoots are very high in sugar. Captive pandas eat ‘panda cake’ biscuits in addition to bamboo so the higher protein content might affect blood parameters. We did not find that to be the case in our study. There are changes in panda metabolomics depending upon diet (Guo *et al.,*2019). Therefore, tracking blood parameter data, along with diet, can be highly informative to the success of conservation translocation programs because they provide baseline levels to which data on released individuals can be compared.

Wild giant panda populations live in subtropical and temperate climate regions in China, while captive individuals are distributed globally in zoos located in Asia, North America and Europe. Wild giant pandas can utilize a wide elevation gradient throughout the year to meet their breeding and resource requirements. Most giant pandas move gradually from relatively low-elevation (1200–1800 m) in the mating season (September to April) upwards to high-elevation bamboo forest in the non-mating season (Pan *et al.,*2014). Our study confirms that the giant panda acclimates to changes in elevation as reflected in physiological blood parameters. This information is essential for understanding the survivability of giant pandas in the wild and allows us to better monitor the health of giant pandas selected for translocation and rewilding.

The elevation and conditions in our enclosures allowed the giant pandas to acclimatize to the elevation as if they were free ranging in the reserves. Therefore, our findings should be predictive for giant pandas when released to live freely in nature. Studies on deer mice have already shown that the mass of heart and lungs increase together with increased blood content under high altitude semi-natural conditions (Hammond *et al.,*1999, 2001). It is unlikely that we will be able to measure organ sizes on living giant pandas in nature, but we can assume that they will undergo similar changes in organs as did those mice.

## Conclusions

Our study was the first study to report hematological and biochemical parameters of the blood of giant pandas that were living in natural conditions in nature reserves. Giant pandas acclimatized physiologically when moved to a large natural high-altitude enclosure from a captive enclosure. Their blood hematology changed, and their blood biochemistry remained normal. The RIs we established for blood parameters of giant pandas based on both the captive and release animals in captivity provide a basis for monitoring the pre- and post-release health status of giant pandas in our soft release and other reintroduction programs. We only had three animals in the release program. Therefore, additional data are needed on blood parameters of release pandas, but those data will only come as more animals go through the soft-release program and make their way into nature reserves.
